# Feasibility and Safety of Rapid Drug Desensitization for Chemotherapy-Induced Maculopapular Eruptions

**DOI:** 10.3390/jcm15166320

**Published:** 2026-08-15

**Authors:** Meryem Demir, Nilay Duman, Haydar Soydaner Karakus, Sercan On, Kasim Okan, Hatice Serpil Akten, Sinem Inan, Su Ozgur, Tuncay Goksel, Erdem Goker, Ozlem Goksel

**Affiliations:** 1 Laboratory of Occupational & Environmental Respiratory Diseases and Asthma, Division of Immunology and Allergy, Department of Pulmonary Medicine, Faculty of Medicine, Ege University, 35100 Izmir, Türkiye; meryem_durgay@hotmail.com (M.D.);; 2 Department of Dermatology, Faculty of Medicine, Ege University, 35100 Izmir, Türkiye; 3 Translational Pulmonary Research Center-EgeSAM, Ege University, 35100 Izmir, Türkiye; 4 Department of Medical Oncology, Faculty of Medicine, Ege University, 35100 Izmir, Türkiye

**Keywords:** antineoplastic agents, delayed hypersensitivity, drug desensitization, drug hypersensitivity, exanthema, imatinib, paclitaxel

## Abstract

**Background/Objectives**: Chemotherapy drug (ChD)-induced maculopapular eruptions (MPEs) may interrupt optimal cancer treatment when no equally effective alternative therapy is available. Evidence supporting rapid drug desensitization (RDD) for delayed-type MPE remains scarce. We aimed to evaluate the feasibility and safety of RDD in patients with ChD-induced MPE. **Methods**: Adult patients with ChD-induced MPE who underwent RDD between January 2021 and January 2024 were evaluated. Delayed MPE recurrence was assessed until the subsequent chemotherapy cycle. **Results**: A total of 63 RDD procedures were performed in 10 patients with ChD-induced MPE, most commonly triggered by taxanes and platinum agents. The median onset of the index reaction was 60.0 h (24.0–336.0), and delayed intradermal tests were positive in 2 of 9 tested patients (22.2%). No delayed MPE recurrences were observed during the predefined follow-up period until the subsequent chemotherapy cycle. Immediate breakthrough reactions occurred in 3 of 10 patients (30.0%), corresponding to 17 of 63 RDD procedures (27.0%), and were limited to mild flushing and pruritus that resolved with antihistamines with or without low-dose corticosteroids. All RDD procedures were successfully completed without severe systemic reactions, and all patients were discharged on the same day. **Conclusions**: In carefully selected patients with delayed-type MPE, RDD appears to be a feasible and safe short-term strategy for enabling continuation of essential chemotherapy within the predefined observation period. As this pilot study includes a small and heterogeneous patient population with limited follow-up, the findings should be considered preliminary and hypothesis-generating and warrant confirmation in larger prospective studies with longer follow-up.

## 1. Introduction

Chemotherapy drugs (ChD) remain the cornerstone of treatment for many malignancies. However, they are also a major cause of hypersensitivity reactions (HSRs) and represent the third-leading cause of fatal drug-induced anaphylaxis in the United States [1]. Over the past two decades, rapid drug desensitization (RDD) has become an established strategy for managing ChD-induced immediate HSRs. The 2022 European Academy of Allergy and Clinical Immunology (EAACI) position paper, an expert consensus document providing evidence-based recommendations on ChD HSRs, recommends RDD not only for immediate HSRs but also for selected delayed type IV HSRs, excluding severe cutaneous adverse reactions (SCARs), while highlighting the need for larger studies to better define its efficacy and safety in delayed HSRs [1].

However, the optimal selection of patients with delayed HSRs who may benefit from RDD remains uncertain, particularly when no equally effective alternative ChD is available. These unresolved issues are further highlighted in the recent World Allergy Organization (WAO) statement on intravenous RDD and delabeling [2].

Maculopapular eruption (MPE) is the most common delayed-type drug-induced HSR [3]. MPE is classified as a type IV T cell-mediated hypersensitivity reaction, typically occurring within 7–12 days of treatment initiation and frequently recurring upon re-exposure due to drug-specific T-cell clones. Histopathology usually reveals superficial perivascular lymphocytic infiltrates with eosinophils. RDD is a promising treatment modality for patients with ChD allergy [4]. Evidence supporting RDD for ChD-induced delayed MPE is limited to small case series [5,6,7,8,9]. Because of its non-life-threatening nature and T cell-mediated pathogenesis, MPE is considered one of the most suitable delayed HSR phenotypes for RDD [10]. Since 2011, we have performed more than 1350 RDD procedures for immediate ChD-induced HSRs and have subsequently extended this approach to carefully selected patients with delayed MPE [11,12,13].

In this study, we aimed to evaluate the feasibility and safety of RDD in patients with ChD-induced delayed MPE. The primary outcome was successful completion of RDD without recurrence of delayed MPE until the subsequent chemotherapy cycle, while secondary outcomes included the incidence, timing, and severity of breakthrough reactions (BTRs) and the overall tolerability of repeated RDD procedures.

## 2. Materials and Methods

### 2.1. Study Design and Patient Selection

This was a single-center, observational clinical study conducted at Ege University Faculty of Medicine, Pulmonary Immunology and Allergy Department, a tertiary severe drug allergy desensitization unit in Izmir, Türkiye. Patients were referred from the medical oncology departments between January 2021 and January 2024, having been diagnosed with ChD-induced delayed-type (>6 h) MPE.

Patients were considered eligible candidates for RDD when all of the following criteria were met:

1. The culprit drug was considered essential for oncologic management;
2. The index reaction was diagnosed as delayed MPE by both an allergist and a dermatologist;
3. Recurrent MPE developed despite intensified premedication during subsequent treatment cycles;
4. No equally effective alternative treatment option was available;
5. Differential diagnoses were excluded;
6. Patients were adults (>18 years of age) and provided written informed consent.

Patients with SCARs, clinically high-risk presentations, or suitable alternative oncologic treatment options were not considered candidates for RDD.

The study was conducted in accordance with the Declaration of Helsinki and approved by the Ethics Committee of Ege University Faculty of Medicine (approval no. Ege21-8.4/36; approval date: January 2021.

### 2.2. Differential Diagnosis of MPE and Identification of the Culprit Drug for RDD

Patients with active infection at the time of MPE, concomitant inflammatory dermatoses, or alternative causes of recurrent skin eruptions were excluded. All patients underwent laboratory evaluation, including complete blood count, biochemical testing, inflammatory markers, and screening for acute or reactivated viral infections. Concomitant medications that had been tolerated before and after resolution of MPE were considered unlikely to be causative. The culprit ChD was identified based on clinical assessment, supported by skin test (ST) and/or skin biopsy when available, together with a history of at least two delayed HSRs under the same agent. When two suspected culprit ChDs had been administered during the same treatment cycle, sequential RDD was performed for both.

### 2.3. Allergy Work-Up

Patients’ baseline atopic status was evaluated by skin prick testing (SPT) using a panel of common aeroallergens, including region-specific pollens, house dust mites, molds, and animal danders (Lofarma Allergeni, Milan, Italy). Baseline total IgE and tryptase levels were recorded. Saline and histamine served as the negative and positive controls, respectively, and a wheal diameter ≥ 3 mm was considered a positive result.

Subsequently, ST with the culprit ChD were performed and interpreted according to the EAACI/ENDA guidelines by Brockow et al. [14]. ChD SPT were performed using the undiluted drug and, if negative, were followed by intradermal tests (IDTs) with serial dilutions. Delayed IDT readings were obtained at 24, 48, and 72 h. ST was performed in nine patients. Patient 3, who received liposomal doxorubicin, was not tested because ST for this agent has not been standardized or validated and may cause local irritative or toxic reactions. ST concentrations are summarized in Table 1.

ST were performed at least 7 days after discontinuation of antihistamines and 2–3 weeks after cessation of systemic corticosteroids. Grading of MPE was performed according to the National Cancer Institute Common Terminology Criteria for Adverse Events (CTCAE), version 5.0. (Table 2) [15].

### 2.4. Premedication Procedure

All patients had received the institutional standard oncologic premedication protocol before the index reaction, consisting of intravenous dexamethasone (8–12 mg), pheniramine, and ondansetron. For cisplatin-containing regimens, aprepitant and palonosetron were added. The same premedication protocol was administered before each RDD procedure. No premedication was given before the index reaction or during RDD with oral imatinib. None of the patients received continuous corticosteroid therapy between RDD procedures.

### 2.5. Rapid Drug Desensitization Procedure

The decision to perform RDD was made jointly by the patient, the treating oncologist, and the allergy team. RDD was initiated only after complete clinical resolution of the index MPE. Therefore, recurrence was defined as the development of a new MPE following RDD and was distinguished from persistence or incomplete resolution of the index eruption.

RDD was performed in the intensive care unit by an experienced multidisciplinary team comprising pharmacists, nurses, intensivists, and allergy specialists. Throughout each RDD procedure, patients were continuously monitored by the nursing staff under the supervision of allergy specialists and intensivists.

A three-solution, 12-step RDD protocol, originally described by Castells et al. and subsequently used in our previous studies, was applied [11,12,13,16]. Patients received the full therapeutic dose of the culprit ChD during each RDD procedure.

Chemotherapy solutions were prepared in a three-bag system using 250 mL dextrose or normal saline. Infusions were initiated at 2 mL/h and advanced every 15 min to a maximum rate of 80 mL/h under continuous vital sign monitoring. Uncomplicated RDD procedures were completed in approximately 6 h. If a BTR occurred, the infusion was interrupted and appropriate treatment was administered before resuming the protocol.

In three patients (Patients 1, 2, and 4), RDD was performed sequentially for two culprit ChDs administered 1–7 days apart. The 8-step oral RDD protocol for imatinib was adapted from previously published protocols and our institutional experience (Table 3) [17].

### 2.6. Statistics

Statistical analyses were performed using IBM SPSS Statistics for Windows, Version 20.0 (IBM Corp., Armonk, NY, USA). Due to the small sample size and exploratory nature of the study, analyses were primarily descriptive. Categorical variables were presented as frequency (n) and percentage (%), whereas continuous variables were expressed as mean ± standard deviation (SD) or median with minimum–maximum values, as appropriate according to data distribution. For proportions, 95% exact binomial confidence intervals were calculated using the Clopper–Pearson method. Because of the limited sample size and absence of a comparator group, no formal comparative or multivariable statistical analyses were performed.

## 3. Results

### 3.1. Demographics and Clinical Characteristics of Patients

A total of 10 patients with ChD-induced MPE underwent 63 RDD procedures. The mean age was 58.1 ± 12.7 years, and eight patients (80.0%) were female. The most common malignancy was lung cancer (40.0%), followed by ovarian and breast cancer (20.0% each), with one patient each diagnosed with cancer of unknown primary and chronic myeloid leukemia (CML). Five of nine patients (55.6%) had stage IV disease. The median Karnofsky Performance Status was 100 (range, 70–100). Median total IgE and tryptase levels were 25.0 kU/L (range, 2.82–507.0) and 5.3 µg/L (range, 2.9–24.0), respectively. Peripheral eosinophil counts, renal function, and liver enzyme levels were within normal limits.

MPE was most frequently associated with platinum- and taxane-based ChD, whereas gemcitabine/cisplatin, liposomal doxorubicin, and oral imatinib were identified as culprit agents in individual patients. The median time to MPE onset was 60.0 h (range, 24.0–336.0), after a median of three prior chemotherapy cycles (range, 2–5). According to CTCAE v5.0, the index reaction was mild in five patients (50.0%), moderate in four (40.0%), and severe in one (10.0%). Patient characteristics and RDD outcomes are summarized in Table 4.

**Figure 1:**
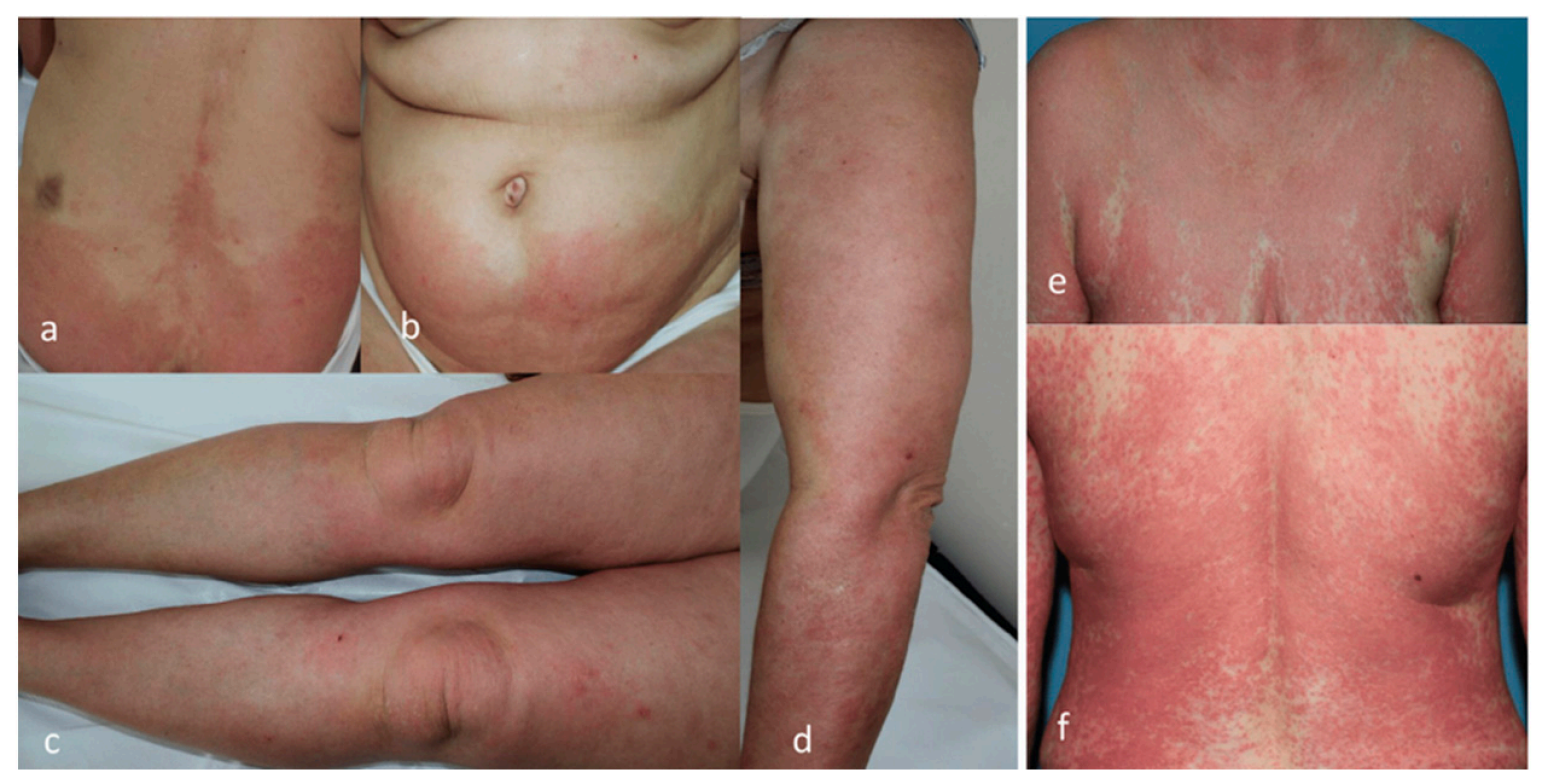
Maculopapular eruption = MPE: The primary lesions are erythematous pink-to-red macules or infiltrated papules. The exanthems predominantly involves the trunk and proximal extremities in a symmetric distribution and usually presents as morbilliform (measles-like) or rubelliform (rubella-like). Mucous membranes are normally not involved. Pruritus is typical. The lesions often blanch with pressure but may be purpuric on the lower legs and they can last days. Continuous exposure to the culprit drug causes the lesions to become persistent. (**a**–**f**): Symmetrically located MPE with coalescence in some areas on the trunk and extremities. The photo was taken with the written consents of the patient 10 treated with imatinib (**a**–**d**), and patient 2 treated with paclitaxel and carboplatin (**e**,**f**).

**Figure 2:**
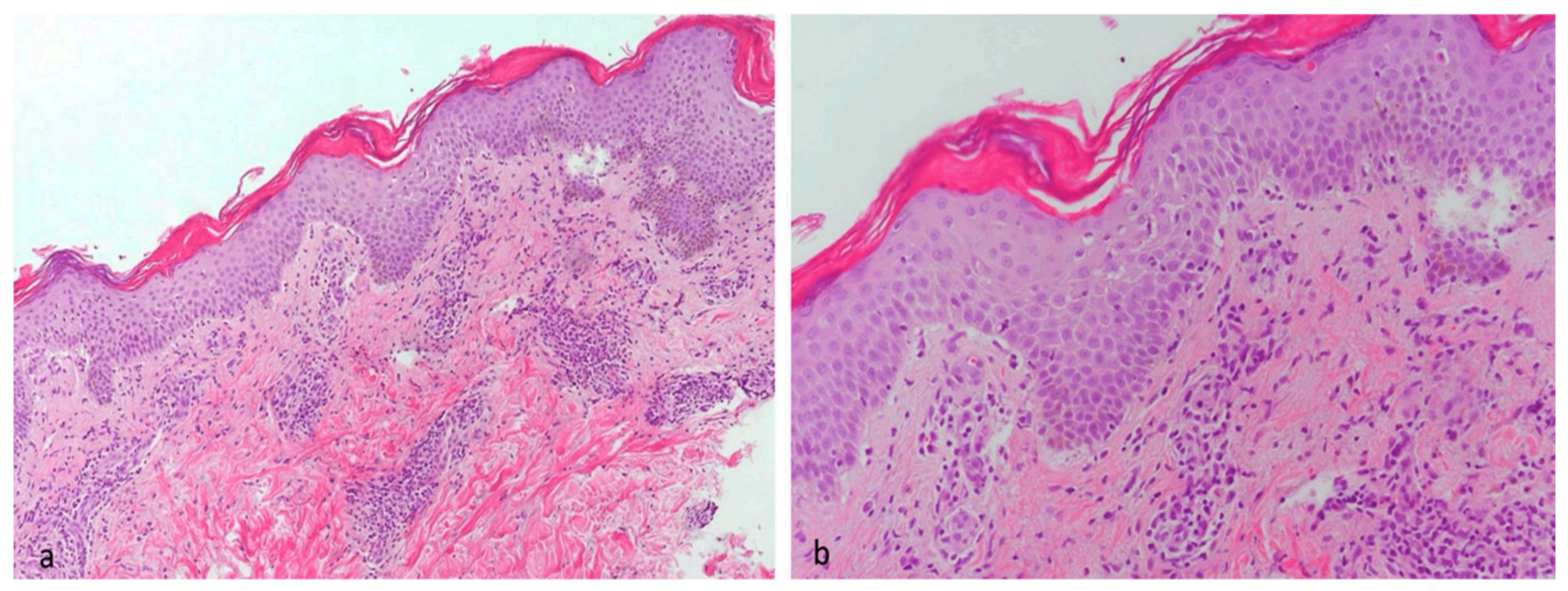
Histopathologic findings of clinically confirmed drug-induced MPE in skin biopsy. The histological features of MPE show a mild vacuolar interface dermatitis with dyskeratotic keratinocytes and superficial, perivascular, lymphocytic infiltrate with scattered eosinophils. ((**a**): H&E ×40, (**b**): H&E ×100). This photomicrograph was obtained from the skin biopsy of Patient 10 with imatinib-induced delayed hypersensitivity and was provided by the Department of Pathology, Ege University Faculty of Medicine.

MPE and histopathologic findings are shown in Figure 1 and Figure 2.

### 3.2. Allergo-Oncological Evaluation

General atopy evaluation with a common prick test panel was negative in all patients. ST (SPT, IDT) with culprit drugs were performed in 9 of the 10 patients, as one patient receiving liposomal doxorubicin was not tested because ST for this agent has not been standardized or validated and may be associated with local irritative or toxic effects. Skin prick tests were negative in all nine patients. However, late IDT at a 1/10 concentration on the 48 h reading was observed in two patients with docetaxel-induced MPE (2/9, 22.2%).

### 3.3. Outcomes of the Rapid Drug Desensitizations and Breakthrough Reactions

A total of 63 RDD procedures were performed, including 27 with oxaliplatin, 14 with carboplatin, 10 with paclitaxel, 5 with docetaxel, 2 each with gemcitabine, cisplatin, and liposomal doxorubicin, and 1 with oral imatinib. Seven patients (70.0%) completed all RDD procedures without BTRs. BTRs occurred during 17 of 63 RDD procedures (27.0%) and in 3 of 10 patients (30.0%). Because multiple RDD procedures were performed in the same patients, cycle-level estimates should be interpreted as descriptive rather than as independent event rates.

In Patient 4, facial erythema and pruritus developed at the final step of all eight RDD procedures. The infusion was temporarily interrupted, intravenous pheniramine (45.5 mg) was administered, and RDD was successfully completed, although mild facial erythema persisted for up to 72 h. Patient 6 experienced recurrent palmar erythema and pruritus during the final step of eight RDD procedures, which were managed similarly, with symptoms resolving within 48 h. Patient 7 developed facial erythema only once at step 8 and successfully completed RDD after antihistamine treatment. No patient required oxygen supplementation, nebulized bronchodilators, or intramuscular epinephrine. All patients were evaluated for MPE recurrence by telephone after RDD and at face-to-face follow-up before the subsequent chemotherapy cycle. All RDD procedures were successfully completed, and all patients were discharged on the same day.

## 4. Discussion

In this study, we report the outcomes of 63 RDD procedures performed in 10 patients with ChD-induced MPE, representing a single-center series focused on delayed-type MPE. All RDD procedures were successfully completed, with no recurrence of MPE during the predefined follow-up period until the subsequent chemotherapy cycle. These findings help address an important evidence gap identified by recent EAACI and WAO position papers regarding delayed HSRs to antineoplastic agents [2]. Although our results suggest that RDD may be a feasible short-term approach for carefully selected patients requiring continuation of the culprit drug, the limited follow-up precludes conclusions regarding sustained clinical tolerance beyond the subsequent chemotherapy cycle.

Although no patient developed a more severe delayed cutaneous reaction than the index MPE, immediate BTRs occurred in three patients during RDD, suggesting a possible ‘converter phenotype’. All reactions were mild, without systemic involvement according to the Brown grading system, and resolved with antihistamines and/or low-dose corticosteroids [18]. Similar findings have been reported by Vega et al., who described 59 RDD procedures in eight patients with delayed cutaneous reactions, predominantly MPE, with a lower BTR rate than observed in our cohort (17.2% vs. 30.0%), while all patients successfully completed treatment [5]. Compared with our findings, the lower BTR rate reported in Vega et al.’s study may be partially explained by differences in patient characteristics and culprit agents. Likewise, successful RDD without significant BTRs has been reported in individual cases of bendamustine-induced MPE [6,7]. Repeated exposure to the culprit drug may result in immediate reactions, a phenomenon described as the “converter phenotype,” which was reported in 10.3% of patients undergoing desensitization to chemotherapy or biologic agents by Jimenez et al. [19].

Evidence for slow desensitization in ChD-induced delayed HSRs is also limited. Demir et al. successfully applied a 16-day desensitization protocol in 10 patients with lenalidomide-induced non-immediate HSRs, including three with MPE [8]. Similarly, Lee et al. reported successful rapid and slow desensitization in five patients with delayed cutaneous reactions to lenalidomide; however, none had MPE, and all presented with mild skin reactions such as urticaria [9].

Platinums and taxanes are among the most common causes of delayed ChD-induced HSRs [20]. Although the incidence of delayed HSRs to platinum agents remains uncertain [21,22,23], neither of our patients with isolated oxaliplatin- or carboplatin-induced delayed HSRs experienced recurrence after 27 and 6 subsequent RDD procedures, respectively. Similarly, Pradelli et al. reported delayed HSRs in 8.4% of patients receiving platinum agents or taxanes, including several cases of MPE, and demonstrated carboplatin–oxaliplatin cross-reactivity on delayed IDT [24].

Imatinib, a tyrosine kinase inhibitor, is commonly associated with MPE as its primary cutaneous adverse reaction [17,25]. In our cohort, an 8-step oral RDD protocol enabled successful continuation of imatinib in one patient with CML without BTRs, and treatment was maintained uneventfully for one year. Similarly, successful slow desensitization for delayed imatinib-induced HSRs has previously been reported [26].

In our cohort two positive delayed readings of IDTs were found with docetaxel (n:2/9, 22.2%) The diagnostic role of delayed ST in ChD-induced delayed HSRs remains incompletely defined. Although positive delayed ST results may indicate immunologic sensitization, they do not necessarily predict clinical recurrence or RDD outcomes, whereas negative results cannot reliably exclude delayed HSRs [27]. In our cohort, neither patient with a positive delayed IDT developed BTRs, whereas all BTRs occurred in patients with negative ST. These findings suggest that delayed IDT results alone may not predict RDD outcomes.

Although the mechanisms of RDD in delayed HSRs are not fully understood, modulation of both innate and adaptive immune responses is thought to contribute to temporary clinical tolerance. Teraki et al. demonstrated reduced lesional CD8+ T cell infiltration and increased CD4 + CD25+ regulatory T cells during allopurinol desensitization, suggesting modulation of T cell-mediated immune responses [28]. In addition, RDD has been associated with increased regulatory cytokines, particularly IL-10, the emergence of IL-35-producing regulatory T cells, and reduced anti-drug antibody levels [29,30].

This study has several limitations. First, the small sample size and heterogeneous patient population limit the generalizability of the findings. Patients differed with respect to tumor type, culprit ChD, and prior chemotherapy exposure, all of which may influence delayed HSRs and response to RDD. Therefore, these findings should be considered proof-of-concept rather than universally applicable to individual ChDs.

Second, because multiple RDD procedures were performed in the same patients, cycle-level observations were not statistically independent, and the majority of procedures were contributed by a small number of patients. The predefined endpoint was limited to the absence of MPE recurrence until the subsequent chemotherapy cycle rather than cumulative long-term risk. Larger prospective studies are needed to validate these findings and account for within-patient correlation.

Third, the single-center design and absence of a control group may have introduced center-specific bias and limit causal inference regarding RDD efficacy. Finally, in vitro diagnostic tests, including lymphocyte transformation tests, as well as detailed immunophenotyping and functional immunological analyses, were not performed, limiting mechanistic interpretation. In addition, RDD was performed in a highly specialized ICU setting with multidisciplinary expertise, which may limit the generalizability of this approach to other centers.

## 5. Conclusions

In carefully selected patients with delayed-type MPE, RDD appears to be a feasible and safe short-term strategy for enabling continuation of essential chemotherapy within the predefined observation period. As this pilot study includes a small and heterogeneous patient population with limited follow-up, the findings should be considered preliminary and hypothesis-generating and warrant confirmation in larger prospective studies with longer follow-up.

## Figures and Tables

**Table 1:** Non-irritating concentrations of skin tests for ChD [1].

	Prick Test Concentrations (mg/mL)	Intradermal Test Concentrations (mg/mL)
Cisplatin	1/1 (0.5)	1/100 (0.005) 1/10 (0.05)
Carboplatin	1/1 (10)	1/100 (0.1) 1/10 (1)
Oxaliplatin	1/1 (5)	1/100 (0.05) 1/10 (0.5)
Paclitaxel	1/1 (6)	1/100 (0.06) 1/10 (0.6)
Docetaxel	1/1 (1)	1/100 (0.01) 1/10 (0.1)
Gemcitabine	1/1 (38)	1/100 (0.038) 1/10 (0.38)

**Table 2:** Grading of MPE according to CTCAE v5.0 [15].

Grade	Severity	Description
1	Mild	Macules/papules covering <10% BSA with or without symptoms (e.g., pruritus, burning, tightness)
2	Moderate	Macules/papules covering 10–30% BSA with or without symptoms (e.g., pruritus, burning, tightness); limiting instrumental ADL, rash covering >30% BSA with or without mild symptoms
3	Severe	Macules/papules covering >30% BSA with moderate or severe symptoms; limiting self-care ADL

ADL: Activities of Daily Living, BSA: Body Surface Area.

**Table 3:** Eight-step desensitization protocol for imatinib [17].

Step	Concentrate	Volume, mL	Dose, mg
1	10 ng/mL	1, 2, 4	0.00007
2	100 ng/mL	1, 2, 4	0.00075
3	1 µg/mL	1, 2, 4	0.0075
4	10 µg/mL	1, 2, 4	0.075
5	100 µg/mL	1, 2, 4	0.75
6	1 mg/mL	1, 2, 4	7.5
7	10 mg/mL	1, 2, 4	75.0
8	100 mg tab	1	100.0

**Table 4:** Characteristics of patients with delayed-type MPE and outcomes of RDD.

	Patient 1	Patient 2	Patient 3	Patient 4	Patient 5	Mean ± SD; Median [Min–Max]
**Age (years)**	62	52	76	39	45	58.1 ± 12.7
**Gender**	Female	Female	Female	Female	Female	80.0% Female
**Cancer type**	Lung Ca	Lung Ca	Ovarian Ca	Ovarian Ca	Breast Ca	40.0% Lung Cancer
**Disease stage**	4	4	4	1	2	55.6% Stage 4
**Number of chemotherapy cycles prior to index reaction**	2	3	2	3	5	3.0 [2.0–5.0]
**Culprit agent**	Gemcitabine/Cisplatin	Paclitaxel/Carboplatin	Liposomal doxorubicin	Carboplatin/Paclitaxel	Paclitaxel	-
**MPE timing (hour)**	24	96	24	48	24	60.0 [24.0–336.0]
**MPE severity** **(BSA %) ^a^**	Mild (<10%)	Severe (>30%)	Moderate (10–30%)	Mild (<10%)	Mild (<10%)	50.0% Mild
**Skin biopsy**	NA	Drug induced MPE	Drug induced MPE	NA	NA	
**Duration of MPE treatment (days)**	3	5	4	3	3	3.0 [3.0–5.0]
**SPT/IDT positivity with culprit drug**	Negative	Negative	Non-applicable	Negative	Negative	2/9 (22.2%)
**RDD agent**	Gemcitabine Cisplatin	Paclitaxel, Carboplatin	Liposomal doxorubicin	Carboplatin, Paclitaxel	Paclitaxel	-
**RDD protocol**	12-step	12-step	12-step	12-step	12-step	
**Total RDD number**	4	8	2	8	2	3.5 [1.0–27.0]
**BTR number**	0	0	0	8	0	-
**BTR type**	-	-	-	Facial hyperemia and itching on step-12 and after 72 h	-	-
**BTR treatment**				Antihistamines on step-12 and for 3 days		-
**Outcome of RDD**	Completed	Completed	Completed	Completed	Completed	-
	**Patient 6**	**Patient 7**	**Patient 8**	**Patient 9**	**Patient 10**	**Mean ± SD;** **Median [Min–Max]**
**Age (years)**	51	75	69	62	50	58.1 ± 12.7
**Gender**	Female	Male	Male	Female	Female	80.0% Female
**Cancer type**	Liver mass	Lung Ca	Lung Ca	Breast Ca	CML	40.0% Lung Cancer
**Disease stage**	2	4	4	2	Non-applicable	55.6% Stage 4
**Number of chemotherapy cycles prior to index reaction**	3	2	3	2	14th day	3.0 [2.0–5.0]
**Culprit agent**	Oxaliplatin	Carboplatin	Docetaxel	Docetaxel	Imatinib	-
**MPE timing (hour)**	48	72	72	96	336	60.0 [24.0–336.0]
**MPE severity** **(BSA %) ^a^**	Moderate (10–30%)	Mild (<10%)	Mild (<10%)	Moderate (10–30%)	Moderate (10–30%)	50.0% Mild
**Skin biopsy**	NA	NA	NA	NA	Drug induced MPE	
**Duration of MPE treatment (days)**	3	3	3	3	3	3.0 [3.0–5.0]
**SPT/IDT positivity with culprit drug**	Negative	Negative	Late IDT positive	Late IDT positive	Negative	2/9 (22.2%)
**RDD agent**	Oxaliplatin	Carboplatin	Docetaxel	Docetaxel	Imatinib	-
**RDD protocol**	12-step	12-step	12-step	12-step	8-step	
**Total RDD number**	27	6	3	2	1	3.5 [1.0–27.0]
**BTR number**	8	1	0	0	0	-
**BTR type**	Palmar hyperemia and itching on step-12 and after 48 h	Facial hyperemia on step-8	-	-	-	-
**BTR treatment**	Antihistamines on step-12 and for 2 days	Antihistamines on step 8				-
**Outcome of RDD**	Completed	Completed	Completed	Completed	Completed	-

BSA: Body Surface Area, BTR: Breakthrough Reaction, Ca: Cancer, ChDs: Chemotherapy Drugs, CML: Chronic Myeloid Leukemia, MPE: Maculopapular Eruption, NA: Non-applicable, RDD: Rapid Drug Desensitization, SPT: Skin Prick Testing, ST: Skin Test (Prick testing + intradermal testing), Rx: Reaction. ^a^ Common terminology criteria for adverse events version 5.0 (CTCAE v5.0).

## Data Availability

The datasets generated and/or analyzed during the current study are not publicly available due to ethical restrictions and the inclusion of potentially identifiable patient information but are available from the corresponding author on reasonable request and with approval of the institutional review board.

## Abbreviations

The following abbreviations are used in this manuscript:

BTR	Breakthrough reaction
ChD	Chemotherapy drugs
CML	Chronic myeloid leukemia
CTCAE	Common Terminology Criteria for Adverse Events
EAACI	European Academy of Allergy and Clinical Immunology
HSR	Hypersensitivity reactions
IDT	Intradermal test
IgE	Immunoglobulin E
MPE	Maculopapular eruption
PT	Patch test
RDD	Rapid drug desensitization
SCARs	Severe cutaneous adverse reactions
SPT	Skin prick testing
ST	Skin test
WAO	World Allergy Organization
